# Randomised clinical trial to evaluate changes in dentine tubule occlusion following 4 weeks use of an occluding toothpaste

**DOI:** 10.1007/s00784-017-2103-5

**Published:** 2017-04-01

**Authors:** Joon Seong, Charles P. Parkinson, Maria Davies, Nicholas C. A. Claydon, Nicola X West

**Affiliations:** 1 Periodontology, Clinical Trials Unit, Bristol Dental School, Lower Maudlin Street, Bristol, BS1 2LY UK; 2 Oral Care Medical Affairs Research & Development, GSK Consumer Healthcare, St Georges Avenue, Weybridge, Surrey, KT13 0DE UK

**Keywords:** Dentine hypersensitivity, Tubule occlusion, Toothpaste, Replica technique

## Abstract

**Objectives:**

The objective of this study is to determine whether a silicone impression material could precisely replicate dentine tubule changes following 4 weeks toothbrushing with occluding or non-occluding toothpaste and whether changes reflected hypersensitivity clinical assessment.

**Materials and methods:**

This was a single site, examiner blind, parallel, two treatment arm, randomised clinical trial. Participants were healthy, ≥18, with ≥1 sensitive tooth with exposed dentine, Schiff sensitivity score ≥2, and patent tubules with dentine occlusion score 4–5 as determined by scanning electron microscopy of replica impressions. Nine participants received Sensodyne® Rapid Relief (occluding toothpaste) and 10 Crest® Decay Prevention (non-occluding toothpaste), and were re-evaluated for sensitivity and occlusion score after two timed minutes and 4 weeks twice-daily home brushing.

**Results:**

Occlusion scores did not correlate significantly with pain scores, but correlations were positive and impressions showed characteristic dentine tubule patency and occlusion. After 4 weeks, thermal VAS was significantly lower than baseline for the non-occluding toothpaste; all other pain scores were significantly lower for both treatments. Dentine occlusion scores also decreased after 4 weeks of either treatment, but did not achieve significance (*p* = 0.0625).

**Conclusions:**

Both toothpastes reduced clinical sensitivity and increased tubule occlusion. It is hypothesised that during impression, taking some material may have sheared off and occluded tubules resulting in false positives.

**Clinical relevance:**

This study has demonstrated that a silicone impression material can accurately replicate the dentine surface to demonstrate dentine tubular occlusion and patency; however, although the association between occlusion and pain score was positive, this technique needs to be refined before use in future studies.

## Introduction

Dentine hypersensitivity (DH) is an unpleasant and relatively common condition in which thermal, evaporative, tactile, osmotic, or chemical stimuli elicit a short sharp pain response [1]. Prevalence figures for DH vary, but a recent European study demonstrated that 42% of 18–35 year olds suffer from the condition [2]. DH has been shown to adversely affect quality of life [3, 4], and individuals may totally avoid known stimuli due to the severity of the pain they experience [5]. DH arises when dentine is exposed, generally at the cervical margin, following gingival recession and removal of cementum, or where non carious cervical lesions form due to removal of enamel and exposure of dentine, predominantly due to erosion and abrasion [6].

Of the three theories put forward to explain how stimuli at the outer surface of dentine elicit a pain response of the pulpal nerves, the hydrodynamic theory first described by Gysi [7] and later expanded on by Brännström [8] is the most widely accepted. In this theory, it is proposed that stimuli such as cold, hot, tactile, or osmotic pressure, when applied to exposed dentine, cause fluid movement within the dentine tubules. This fluid movement is thought to activate mechano-receptors near the base of the tubules and, if certain physiological parameters are met, result in the firing of an action potential and the generation of a pain response. In support of this theory, it has been demonstrated that it is not sufficient for dentine to simply be exposed to the oral cavity; tubules must also be patent from the pulp to the oral environment [9]. These findings were supported by a study in which it was demonstrated that as compared to normal teeth, dentine tubule orifices of hypersensitive teeth were approximately twice as wide and more densely packed [10]. This study provided the first observation of a relationship between patent dentine and the pain associated with DH.

Based on these studies and the general acceptance of the hydrodynamic theory as the mechanism by which external stimuli cross dentine and elicit a pulpal nerve pain response, the majority of recently developed treatments to alleviate DH have aimed to block dentine tubules [11]. However, while the studies testing the efficacy of such products often employ in situ techniques to demonstrate the occlusion of dentine tubules [12, 13], there is only one published clinical study exploring the impact of an occlusion based therapeutic treatment (Nd:YAG laser) on dentine tubule occlusion and patency, using an impression technique; however, this study did not evaluate whether the occlusion achieved provided relief from the pain of DH [14].

Replica techniques, utilising an impression of the tooth surface together with scanning electron microscopy (SEM) imaging, have been used previously to gain an inverse image of the tooth surface, facilitating visualisation of the areas of DH. It has been demonstrated that it is possible to replicate the tooth surface using a silicone rubber impression material [15] and correlate areas of agglutinated or enlarged dentine tubules visible on replicas with pain response in vivo using an addition polymerisation type of vinyl silicone impression material [16]. Further, the replica technique was also used in a study that demonstrated that the smear layer was often not present in areas of hypersensitivity [17]. These studies have provided valuable information, but no study to date has evaluated whether it is possible to use the replica technique to determine the levels of tubule occlusion necessary to attenuate the pain response and the efficacy of DH treatment toothpastes to alleviate pain.

With the development of new impression materials capable of reproducing submicrometer structure, it is now possible to acquire higher resolution negative images of the surface of dentine, which are both accurate and reproducible under orally relevant conditions. Aquasil impression material utilises the superior properties of both polyethers and additional curing silicones. These impression materials minimise voids and bubbles being hydrophilic and are able to capture an accurate impression of the tooth surface without the need to over-dry the tooth. The material portrays excellent reproduction of detail and good dimensional stability with snap set characteristics, high tear strength, and no swelling or shrinkage. It has all the advantages of a traditional vinyl polysiloxane, such as easier removal from the mouth, no taste, no smell, and the ability to be disinfected/sterilised. In addition, these materials can be imaged directly without the need to cast positive replicas. In 2002, Pereira et al. [18] demonstrated that Aquasil ULV® which in addition to the properties outlined above has a small particle size and was able to reproduce the characteristics of dentine disc surfaces that had been treated with desensitising toothpastes in vitro. Similarly, a study [19] showcased a new impression material that could reproduce and quantify the degree of dentine tubule occlusion afforded by occluding toothpaste in situ. Further, in a second in situ, study it was demonstrated that it was possible to visualise and assess the degree of tubule occlusion on replicas that had been exposed to DH treatments [20]. However, to date, none of these studies, using the more recently developed and more accurate impression materials, have tested them on natural teeth in vivo or tried to relate tubule occlusion scores from replicas with in vivo pain scores.

The present study extends the replica methodology technique, using the new generation impression materials, aiming to correlate clinically diagnosed sensitive or non-sensitive dentine on vital teeth with the surface characteristics of the tooth, i.e. patent or occluded dentine tubules. Changes occurring on the surface of the sensitive dentine following 4 weeks at home use of twice-daily tooth brushing with a toothpaste designed to reduce dentine hypersensitivity by occluding open dentine tubules or a negative control paste known for its non-occluding properties were assessed, and the relationship between changes in the morphology of the dentine surface and the clinical assessment of dentinal hypersensitivity will be explored. It is hypothesised that DH pain scores will correlate with tubule occlusion as assessed by imaging replica impressions.

## Materials and methods

### Study design and methodology

This study was a single site, blind with respect to the study analyst and clinical examiner, parallel, two treatment arm, randomised clinical trial. The study was conducted in a UK Dental School. NHS Research Ethics Committee approval was obtained, and the study was conducted to Good Clinical Practice guidelines as laid down by the Declaration of Helsinki and its later amendments.

Participants aged 18 or over were invited to attend a screening visit, where those happy to take part in the study gave informed consent. Eligibility for inclusion in the study was determined following an oral soft tissue (OST) examination, evaluation of inclusion and exclusion criteria, and the identification of eligible teeth. To be eligible for the study, participants had to be in good general health and have at least one sensitive tooth. Eligible teeth were those with healthy gingivae, but signs of exposed dentine at the cervical margin, that gave an evaporative air (1 s air blast, 21 ± 5 °C) Schiff sensitivity score of 2 or 3 and an occlusion score of 4–5 (Table 1) as determined by SEM of replica impressions of the sensitive area. Impressions of up to four teeth with a Schiff score of >1 were taken per participant using the silicone impression material Aquasil Ultra XLV®, [Dentsply Caulk, USA], and one tooth with the highest occlusion and sensitivity scores was selected for further study. Exclusion criteria included the taking of medicines known to interfere with the perception of pain (such as anticonvulsants, antihistamines, antidepressants, sedatives, tranquillisers, anti-inflammatory drugs, or daily analgesics), tooth bleaching in the past 2 months, and poor oral health. Participants who satisfied all the eligibility requirements at screening were given a standard toothbrush [Aquafresh® Clean Control Medium Toothbrush (GSK Consumer Healthcare, UK)] and washout toothpaste [Crest® Decay Prevention Toothpaste (Procter and Gamble, UK)] for their twice-daily oral hygiene during the washout period between screening and the morning of the baseline visit (2–14 days).

**Table 1:** Scoring systems

Examiners Schiff score	
0	Subject does not respond to air stimulation
1	Subject responds to air stimulus but does not request discontinuation of stimulus
2	Subject responds to air stimulus and requests discontinuation or moves from stimulus
3	Subject responds to stimulus, considers stimulus to be painful and requests discontinuation of the stimulus
Occlusion score	
0	Not evaluable
1	Occluded
2	Mostly occluded
3	Equally occluded/unoccluded
4	Mostly unoccluded
5	Unoccluded

At the baseline visit, participants returned to the study site having brushed their teeth with the washout toothpaste between 1 and 3 h prior to their scheduled visit and having refrained from eating and drinking, with the exception of a limited quantity of water, for at least an hour before their appointment. Participants undertook a VAS training exercise so that they could rate the intensity of their response to stimuli using a 100-mm VAS. Ongoing eligibility was confirmed following an OST examination, accompanied with an assessment of sensitivity of the selected tooth in response to an evaporative air and thermal (ice probe, 1 s application) stimulus as measured by Schiff sensitivity score and VAS, and occlusion score of the sensitive area. For progression into the treatment phase, the tooth selected at baseline had to demonstrate the same minimum Schiff sensitivity and occlusion scores as required for eligibility at the screening visit. Participants who remained eligible for the treatment phase (19 of 20) were randomised by study staff to one of two study treatments according to the computer generated randomisation schedule provided by the study statistician. Randomisation numbers were assigned in ascending numerical order according to appearance at the study site on the day subjects were randomised (baseline visit). Study treatments were a toothpaste designed to reduce dentine hypersensitivity by occluding open dentine tubules [8% strontium acetate toothpaste, 1040 ppm fluoride, as sodium fluoride (Sensodyne® Rapid Relief; GSK Consumer Healthcare, UK)] or a negative control paste known for its non-occluding properties [1450 ppm fluoride, as sodium fluoride (Crest® Decay Prevention Toothpaste; Procter and Gamble, UK)].

Following randomisation, participants were given their treatment toothpaste and a new toothbrush, shown what was meant by a ‘full ribbon’ of toothpaste for their twice-daily home brushing, and supervised during a timed 2-min brushing at the study site. A further assessment of tooth sensitivity using the Schiff sensitivity score and VAS of the selected tooth following evaporative air and thermal ice probe stimuli was conducted, and an impression of the sensitive area of the selected tooth taken within 10 min of product use. On completion of the baseline visit, participants brushed at home, with the provided treatment toothpaste and standard toothbrush for two timed minutes, twice-daily (morning/evening) for 4 weeks (including weekends). During this time, participants were not allowed to use any medication that might affect pain perception or any oral care products except those supplied by the study site with the exception of floss that was permitted for the removal of impacted food.

Participants returned to the study site after 4 weeks having brushed their teeth with their treatment toothpaste between 1 and 3 h prior to their scheduled visit and having refrained from eating and drinking, with the exception of a limited quantity of water, for at least an hour before their appointment. Participants undertook VAS refresher training, and then the sensitivity of the selected tooth in response to evaporative air and thermal (ice probe) was assessed by VAS and Schiff score. Following the sensitivity scores, a final impression of the sensitive area of the selected test tooth was taken.

### Replica impressions

**Fig. 1:**
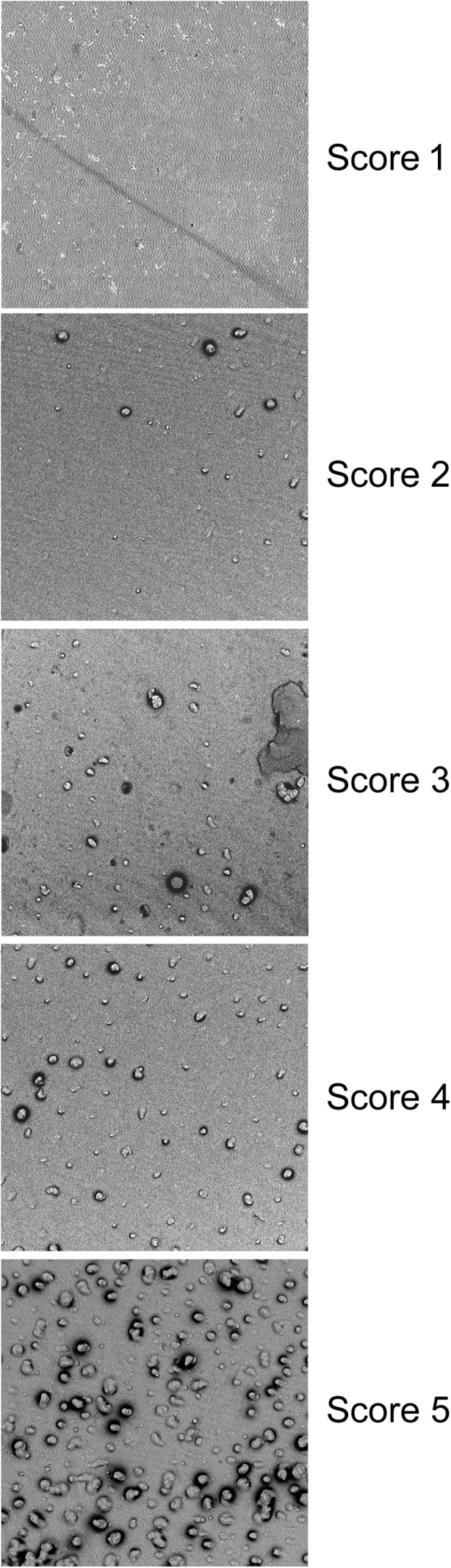
Assessment of occlusion scores**.** Replica images representative of each occlusion score; score 1, fully occluded; score 2, mostly occluded; score 3, equally occluded/unoccluded; score 4, mostly unoccluded; score 5, unoccluded. As images are of negative replicas of the dentine surface, the presence of projections indicates that dentine tubules are unoccluded

Throughout the study, dentine impressions (replicas) were obtained immediately following the clinical sensitivity assessment. Prior to obtaining the replica impression, the surface of the selected tooth was wiped using a damp cotton wool roll, with care taken to ensure no cotton was left on the tooth surface and the impression taken immediately. When taking the impression, the silicone based impression material was applied directly to the tooth surface and held in place for 5 min as instructed by the manufacturer. As dentine in vivo is often affected by intra-oral physical and chemical insults, the surface may be damaged, therefore replicas of up to four sensitive teeth per participant were obtained at screening and baseline so that it was possible to select the tooth on which patent dentine tubules could clearly be observed for further study. Only one replica impression per selected tooth was taken at each study time point. Prior to SEM analysis, replica impressions were disinfected in a solution containing 1000 ppm available chlorine for 10 min, then removed and rinsed well under running water. The replica impression of the sensitive area was analysed directly via SEM without the need to cast a further positive replica at ×2000 magnification using a Phenom benchtop scanning electron microscope (Model Number: 800 03103-02, Phenom-World, The Netherlands) to investigate the degree of dentine tubule occlusion. When capturing the baseline image, a large area of the tooth surface close to the gingival margin was scanned so that the best area possible, where dentine damage was minimal and dentine tubules were clearly patent, could be captured. As well as capturing an SEM image, a light microscope image of the area where open dentine tubules were visible at baseline was taken. Using this image, it was possible to return to the same area of the tooth for the after treatment time points, and using the gingival margin as a reference to ensure that approximately the same location of the replica impression of the tooth was examined on each occasion. Tubule occlusion was scored according to 5-point categorical scale (Table 1, Fig. 1). The SEM imaging and classification was carried out by a single appropriately trained staff member (examiner) who was blind to the treatment that had been applied to the tooth from which the replica impression had been obtained. Before classification of study images, a calibration exercise was performed for the scoring (classification) of replica dentine tubule occlusion SEM images. The examiner graded a standard set of 25 replica dentine tubule occlusion SEM images using the classification grades (Table 1), and the results were compared to the calibrated standard scores for these images [20]. A weighted Kappa coefficient (*κ*) using the Fleiss-Cohen method of weighting [21] where *κ* = 1.0 indicates perfect agreement, and *κ* < 0, no more agreement than would be expected by chance was calculated to assess examiner reliability and reliability was deemed excellent (*κ* > 0.75). Once the examiner had demonstrated acceptable agreement with the calibrated standard, they were approved to classify the study images. At screening and baseline, 37 out of 38 occlusion scores of replica impressions were 5 (unoccluded), demonstrating that oral debris such as salivary deposits which were undoubtedly present did not cause sufficient tubule occlusion to be visible on replica impressions and cause reductions in occlusion score.

### Statistical analysis

This was an exploratory study; the sample size was not based upon statistically powered sample size calculations to detect clinically relevant differences between treatment groups but was considered adequate to provide useful information for the design of future studies. It was anticipated that a maximum of 20 subjects who fulfilled all the entry criteria would be randomised which should ensure that at least 15 evaluable subjects completed the study.

Changes in dentine tubule occlusion scores following a single application of treatment product on day 1 and after 4 weeks were analysed using a Wilcoxon rank sum test to determine differences between treatments and a Wilcoxon signed rank test to assess changes from baseline within treatments.

The change from baseline in the VAS score for the two sensitivity measurements were analysed using an Analysis of Covariance (ANCOVA) model with treatment as fixed effect and the corresponding baseline sensitivity measure (evaporative air VAS or thermal VAS, respectively) as covariate. The assumptions of normality and homogeneity of variance were checked for the ANCOVA model, and no violations were observed.

The difference between treatments with respect to the change from baseline in Schiff scores for the two sensitivity measurements were analysed using a Wilcoxon rank sum test, and a Wilcoxon signed rank test was also performed to look at changes from baseline within treatments.

The number and percentage of participants with decrease, no change, and increase in dentine occlusion score, Schiff score, and VAS following the day 1 application and following 4 weeks of treatment product use for each treatment were summarised. The Fishers exact test was used to assess the difference between treatments.

The relationship between the dentine occlusion tubule score and VAS was summarised using the Pearson’s correlation coefficient for each time point and overall. Similarly, the relationship between tubule occlusion score and Schiff score was assessed for each time point and overall using the Pearson’s correlation coefficient.

## Results

A total of 19 participants were randomised in this study, 9 to the occluding toothpaste group (Sensodyne® Rapid Relief (SRR) and 10 to the non-occluding toothpaste Crest® Decay Prevention (CDP). All participants completed the 4 weeks of treatment. The study was undertaken between April and June 2011. There were 18 female and 1 male participants, with an average age of 43.5. One participant was Asian and the remainder White. Treatment groups were comparable with respect to demographic data. There were no protocol deviations during the study; therefore, the intention-to-treat (ITT) population was the same at the per-protocol (PP) population. There were no treatment emergent adverse events reported.

To determine whether dentine hypersensitivity pain scores correlated with dentine tubule occlusion, all the data from each treatment arm was considered together. When data from all time points for patient report evaporative air VAS and thermal VAS scores were correlated with tubule occlusion, there were no significant correlations (Pearson’s correlations of 0.2145 and 0.1259, respectively). Similarly, when data from all time points for examiner reported evaporative air Schiff and thermal Schiff scores were correlated with tubule occlusion, there were no significant correlations (Pearson’s correlations of 0.2704 and 0.2367, respectively). Although correlations were not significant, the correlation observed was positive and suggested that both VAS and Schiff scores increased as tubule occlusion decreased.

**Fig. 2:**
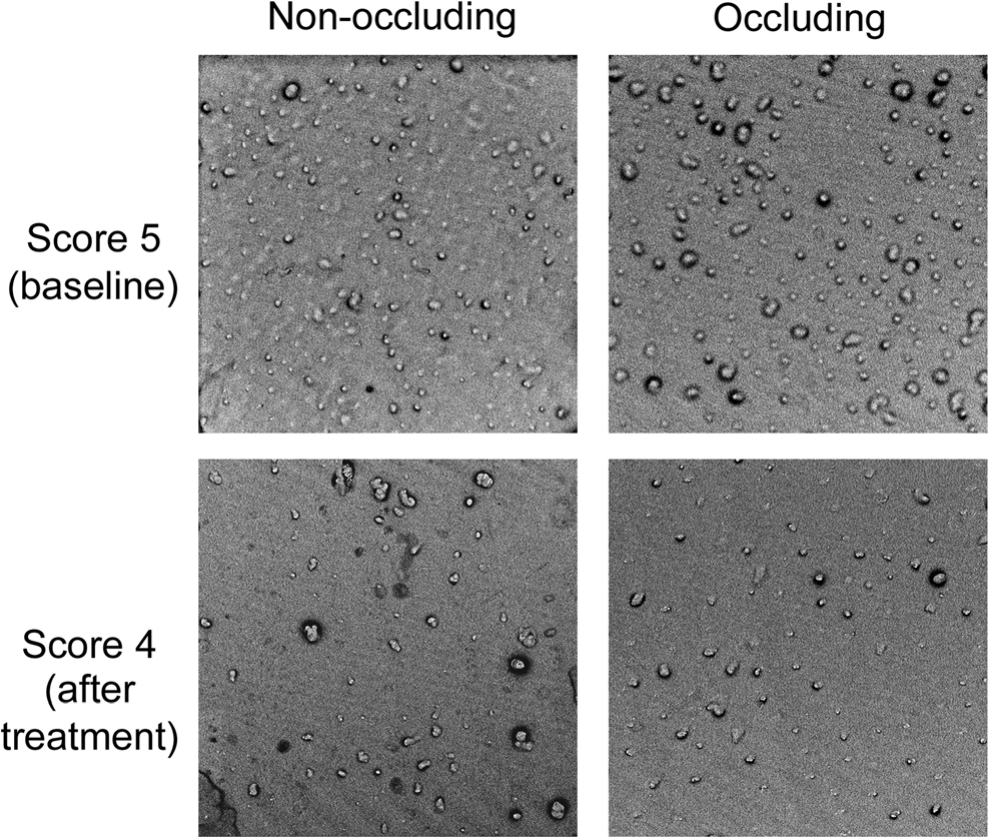
Representative images of replicas before and after treatment. Images of replicas taken at baseline and after treatment, with some evidence of impression material shearing evident, where projections are visible, but do not protrude far from the surface. Images were captured at ×2000 magnification

Analysis within treatments demonstrated that after 4 weeks of treatment, sensitivity scores decreased significantly for both Sensodyne® Rapid Relief (SRR) and Crest® Decay Prevention (CDP) for Schiff sensitivity (*p* < 0.01, SRR; *p* < 0.005, CDP), VAS for evaporative air (*p* < 0.01, SRR; *p* < 0.00001, CDP), and Schiff sensitivity for thermal ice probe (*p* < 0.05 SRR; *p* < 0.01, CDP) (Table 2). Thermal sensitivity as determined by VAS after 4 weeks was only significantly decreased from baseline for the non-occluding toothpaste (*p* < 0.00001). Similarly, occlusion scores fell markedly from baseline; however, the decrease was not significant for either treatment (*p* = 0.0625 for both treatments; Fig. 2). There was no significant change from baseline immediately after first product use (supervised timed 2 min brushing undertaken after randomisation) for either treatment.

**Table 2:** Within treatment summary of baseline, baseline post-treatment and week 4 scores of dentine tubule occlusion, evaporative air VAS, evaporative air Schiff sensitivity and thermal Schiff sensitivity

Efficacy variable	Sensodyne® Rapid Relief (occluding)				Crest® Decay Prevention (non-occluding)			
	No of subjects	Day 1 baseline	Day 1 post-treatment	Week 4	No of subjects	Day 1 baseline	Day 1 post-treatment	Week 4
		Adj. mean, mean or median (*p* value)				Adj. mean, mean or median (*p* value)		
Dentine tubule occlusion score	9	5.0	5.0 (0.5000)	4.0 (0.0625)	10	5.0	5.0 (0.2500)	4.5 (0.0625)
Evaporative air VAS (mm)	9	40.6	39.5 (0.8835)	24.1 (0.0034)	10	34.6	22.4 (0.1007)	9.7 (<0.00001)
Thermal VAS (mm)	9	69.1	71.3 (0.7121)	60.1 (0.1861)	10	76.9	67.6 (0.1054)	42 (<0.00001)
Evaporative air Schiff sensitivity score	9	2	2 (0.4375)	1 (0.0087)	10	2	2 (0.1250)	0 (0.0020)
Thermal Schiff sensitivity score	9	3	3 (1.0000)	2 (0.0313)	10	3	3 (0.6250)	1.5 (0.0039)

Comparing treatments at each time point (Table 3), no statistically significant differences between the two toothpaste treatments were observed after first product use (day 1) for any of the efficacy variables. However, the observed trends (less sensitivity reported) were in favour of the non-occluding toothpaste. After 4 weeks of treatment, the only statistically significant difference between treatments was the change from baseline in thermal VAS (difference of 25.9 mm between treatments, *p* = 0.0114) in favour of the non-occluding toothpaste (Table 3). Overall, the trends observed in the study data appeared to favour the non-occluding toothpaste, with the exception of the dentine tubule occlusion after 4 weeks of treatment where the occluding toothpaste had a greater observed median reduction in dentine tubule patency (1.0) than the non-occluding toothpaste (0.5); however, this difference was not significant (Table 2).

**Table 3:** Between treatment comparison for the changes from baseline in the dentine tubule occlusion score, the evaporative air VAS (mm), the thermal VAS (mm), the evaporative air Schiff sensitivity score, and the thermal Schiff sensitivity score

Efficacy variable	Post-treatment time point	Difference	95% confidence interval for the difference estimate	*p* value
Dentine tubule occlusion score	Day 1	0.0	(0.0, 1.0)	0.6749
Dentine tubule occlusion score	Week 4	0.0	(−3.0, 1.0)	0.6649
Evaporative air VAS (mm)	Day 1	11.1	(−10.6, 32.8)	0.2933
Evaporative air VAS (mm)	Week 4	8.4	(−5.7, 22.5)	0.2245
Thermal VAS (mm)	Day 1	11.5	(−5.5, 28.5)	0.1698
Thermal VAS (mm)	Week 4	25.9	(6.7, 45.1)	0.0114
Evaporative air Schiff sensitivity score	Day 1	0.0	(−1.0, 1.0)	0.4917
Evaporative air Schiff sensitivity score	Week 4	0.0	(0.0, 1.0)	0.2948
Thermal Schiff sensitivity score	Day 1	0.0	(0.0, 1.0)	0.6068
Thermal Schiff sensitivity score	Week 4	1.0	(0.0, 2.0)	0.1385

## Discussion

This study sought to develop an accurate method of quantifying the degree of dentine tubule occlusion using replica technology and to determine how this related to clinical assessments of dentine hypersensitivity pain following treatment with an occluding paste (Sensodyne® Rapid Relief; 8% strontium acetate, 1040 ppm fluoride) and a paste with non-occluding properties (Crest® Decay Prevention; 1450 ppm fluoride) to reduce the pain of DH.

In the current study, dentine occlusion scores did not correlate significantly with clinical pain scores as determined by patient reported VAS and clinician reported Schiff score for either toothpaste tested, although the correlation between occlusion and pain score was positive, thus the study hypothesis is rejected. However, as the correlation was positive, further studies are warranted. Occlusion scores decreased for both treatments to a similar degree but did not reach significance. The decrease in occlusion score (the lower the score the more occluded the dentine tubules) was expected for the occluding toothpaste which has been demonstrated to plug dentine tubules in vitro [22] and occlude tubules in in situ studies [23, 24]. However, in contrast to the current study, in the above in situ studies, treatment with the occluding toothpaste resulted in more tubule occlusion than control toothpastes containing fluoride [23, 24]. Similar to the tubule occlusion findings, in the present study, it was demonstrated that after 4 weeks both the occluding and non-occluding toothpastes reduced Schiff sensitivity score and VAS following stimulation with an evaporative air blast, and Schiff sensitivity score following stimulation with an ice probe, but in contrast to occlusion scores, the decrease in these pain scores was significant. Furthermore, only the non-occluding toothpaste significantly reduced VAS following stimulation with an ice probe. These results are contrary to expectations. The negative control did not contain a specific ingredient designed to provide relief from dentine hypersensitivity by occlusion of patent dentine tubules, clinical studies employing a similar negative control not demonstrating a clinically relevant change from baseline [25, 26]. By contrast, previous clinical studies have demonstrated that the occluding toothpaste used in this study reduced tooth sensitivity in the short and longer term more than a fluoride control toothpaste [25, 26] and to a similar level to two other occluding toothpastes [27].

The current study was not powered to detect difference in treatments but was designed to determine whether a new generation impression material accurately and reproducibly generated a replica of the surface of dentine with enough definition to be able to score the different levels of dentine occlusion resulting from treatments with occluding and non-occluding toothpastes, and relate occlusion scores to pain scores. A small sample size may account for the lack of differences seen for the majority of parameters tested; however, another possible explanation for the findings is that of the observer effect. The observer effect is a phenomenon in which one or more of the techniques used to gather measurements alter the state of the phenomenon that is being measured [28, 29]. In the present study, a small degree of occlusion and surface deposit were visualised following treatment of samples with the non-occluding toothpaste due to some abrasive particles lodging in the tubules and general oral surface deposit as has been seen in a previous study [23]. In addition, the impression material used in this study could penetrate dentine tubules, a necessary characteristic if the degree of tubule occlusion is to be reproduced. However, with the small diameter of the tubules, the penetrating material may have sheared off from the body of the impression during its removal, and the impression material itself at the surface or deeper in the tubule could be occluding the dentine tubules for both treatment groups. This observer effect would occur irrespective of treatment group and could, therefore, result in a false positive trend for efficacy of the control non-occluding product.

As well as potentially causing shearing of the impression material, the relatively small size of dentinal tubules observed in teeth was identified as sensitive in vivo; together with other differences between in vivo and in situ study design, considerations might account for the lack of significant correlation between tubule occlusion and pain score. It was noticeable that the degree of occlusion achieved by the occluding toothpaste in vivo (4.0) was less than the occlusion scores achieved previously in situ by this toothpaste of 2.82 [23] and <2.0 [24]. Greater occlusion may have been achieved in situ due to the protection from abrasion offered by the appliance in which the samples are mounted. However, the degree of tubule occlusion achieved in vivo in the present study was accompanied by a significant reduction in pain score. When the patency of dentine tubules was first correlated with pain score, it was demonstrated that areas of sensitivity had eight times as many open tubules per unit area than areas of non-sensitive dentine [10]. It was also shown that small patent tubules were present in teeth without dentine hypersensitivity, data that suggests in vivo full occlusion of dentine tubules is not required for a tooth to be non-sensitive. In the present study, a scale of 1 (occluded) to 5 (unoccluded) to score the degree of tubule occlusion was used. This scale is commonly used for in situ studies [24, 25] where a broad range of occlusion scores are achieved, perhaps as a result of study design, sample dentine tubule size and density, and the protection afforded by the oral appliance to treated samples. The results of the present study suggest that this scale may be unrepresentative of occlusion and the efficacy of oral products in vivo. A scale for use in vivo with more points between equally occluded/unoccluded and unoccluded might better correlate with pain scores, although examiner scoring on such a scale might prove too challenging.

The finding that it was possible to distinguish different levels of dentine tubule occlusion on replicas taken on natural teeth in vivo, and the relative ease with which the SEM image analyst was able to identify and return to the area of DH on each successive replica suggest that this technique, using impression materials with small particle size, will prove valuable in relating tubule occlusion to pain score in the future. Aquasil® has been used to examine early erosion of enamel in vivo with excellent success of accuracy and reproducibility [30]. However, the technique by which the impression is removed from the mouth requires refinement given the indications that material can shear off from the main body of the impression. Leaving the impression material in place for longer than manufacturers’ recommended time may improve its strength particularly where it projects into the dentine tubules; alternatively, other impression materials may be better suited to this technique and could be tested for efficacy. If the observer effects can be overcome using this technique, it should be possible to see differences in tubule occlusion and relate these to differences in pain scores achieved following treatment with occluding versus non-occluding toothpaste for people with DH. An improved scale with more points to separate degrees of occlusion should be tested for reproducibility, and increasing sample size in future studies will also improve the chances of differences between treatments being significant.

## Conclusions

In summary, this study was unable to confirm that this methodology was suitable for testing product efficacy for DH and could not be used to clarify the relationship between dentine tubule occlusion and clinical pain measurements. More work is needed to clearly demonstrate its value. However, the impression material was able to reproduce different degrees of tubule occlusion in the clinical environment and it was easy to repeatedly revisit designated areas on the tooth surface, suggesting that this method may have utility of tracking tubule patency of time, if refined.
